# Advanced optical phantom mimicking microvascular and directed blood flow in mouse brain

**DOI:** 10.1007/s13534-025-00508-1

**Published:** 2025-09-17

**Authors:** Oleksii Sieryi, Anton Sdobnov, Igor Meglinski, Alexander Bykov

**Affiliations:** 1 Optoelectronics and Measurement Techniques, University of Oulu, 90570 Oulu, Finland; 2 College of Engineering and Physical Sciences, Aston University, Birmingham, B4 7ET UK

**Keywords:** Dynamic optical phantom, Mouse brain, Hemodynamics, Laser speckle contrast imaging

## Abstract

The accurate replication of cerebral hemodynamics is essential for advancing neuroimaging techniques and preclinical research. This study presents a novel multi-component dynamic optical phantom designed to model the complex blood flow dynamics of the mouse brain. The phantom incorporates a static base mimicking skull optical properties, a porous medium infused with a blood-mimicking solution to simulate microvascular perfusion, and a directed flow channel representing large vessels such as the sagittal sinus. The phantom structure was characterized using laser speckle contrast imaging (LSCI) to assess its ability to replicate in vivo-like blood flow patterns. The results demonstrate strong quantitative agreement between the phantom and transcranial LSCI measurements of mouse brain hemodynamics. Our key findings highlight the influence of tissue-mimicking perfusion structures and optical attenuation properties on the blood flow index, validating the phantom as a reproducible and physiologically relevant model. This optically tunable and dynamically controllable platform provides a robust tool for calibrating neuroimaging technologies, validating new optical diagnostic techniques, and investigating cerebral blood flow regulation in preclinical studies.

## Introduction

Optical tissue phantoms are crucial tools in biomedical research, particularly in the fields of optical imaging and sensing [1–3]. The phantoms are artificial models that are intended to mimic optical, mechanical, as well as dynamic properties of real biological tissues, such as a mouse brain, allowing researchers to conduct experiments and calibrations without using actual animal tissues. Phantoms provide a consistent and reproducible medium to calibrate imaging systems or validate new imaging/sensing techniques and algorithms, ensuring accuracy across different experiments and setups.

Phantoms designed to mimic brain tissue must reproduce the unique scattering and absorption characteristics of the brain to ensure that experimental data correspond closely to physiological conditions. Numerous studies [4–7] have focused on developing optical tissue phantoms for human skin, muscle, and blood, often utilizing materials such as silicone, agarose gels, or polyurethane embedded with scattering particles or absorbers like ink and dyes. Additionally, several foundational works published in the *Special Section Guest Editorial* [8] have significantly contributed to advancements in this field.

However, the complex architecture and cellular composition of brain tissue present unique challenges in phantom design.

Authors such as Pogue, Hacker and Christie introduced [1, 9–11] a comprehensive framework for tissue phantoms, detailing material properties, structural characteristics, manufacturing methodologies and methods for matching scattering and absorption properties of biological tissues using organic and synthetic materials.

Researchers [12–14] have also developed layered phantoms to simulate the brain's structural complexity, which includes white matter, gray matter, and cerebrospinal fluid. This layered approach more closely replicates the heterogeneous nature of brain tissue, where each layer contributes differently to light scattering and absorption.

Additionally, the development of 3D-printed phantoms [15–18] has opened new avenues, allowing for the design of phantoms with high spatial accuracy and reproducibility, matching patient-specific anatomical details.

While significant progress has been made in replicating the static optical properties of tissues [13, 19, 20], attention to dynamic components such as blood flow and tissue perfusion has only recently begun to gain focus [21–23], despite their crucial role in simulating cerebral hemodynamics and physiological processes [1]. For example, dynamic optical phantoms incorporating flow channels filled with scattering and absorbing fluids have been introduced [23, 24] to mimic blood flow in the brain. These advanced phantoms enable researchers to study the interaction of light with moving scatterers, providing critical insights into techniques like diffuse correlation spectroscopy (DCS), laser speckle contrast imaging (LSCI) and optical coherence tomography (OCT) for assessing blood flow and oxygenation levels.

Dynamic light scattering based techniques are commonly used for brain imaging because they provide noninvasive monitoring and analysis of cerebral blood flow and microcirculation in real-time [25, 26]. These methods are particularly important for understanding neurovascular dynamics, aiding in the exploration of various physiological and pathological conditions within the brain.

LSCI, a dynamic light scattering (DLS)-based technique, stands out for its ability to capture hemodynamic changes in real-time while maintaining simplicity and accessibility for both clinical and research applications [27–35]. Its non-invasive nature and quantitative capability make it a valuable tool for studying cerebral blood flow dynamics and assessing physiological and pathological conditions in neurovascular research.

LSCI is especially valuable for non-invasive monitoring of cerebral blood flow during surgeries. Its ability to quantitatively analyze blood flow dynamics plays an essential role in advancing our understanding of cerebral hemodynamics, providing a comprehensive perspective on brain function and health.

In this paper, we introduce a practical and easily implementable model of a mouse brain phantom designed to mimic blood-perfused regions, incorporating both directed flow in the sagittal sinus and dynamic perfusion in small multidirectional vessels. The developed multi-component phantom was systematically characterized and validated using LSCI, demonstrating its effectiveness in replicating cerebral hemodynamics for neuroimaging applications.

## Materials and methods

### Phantom preparation

**Fig. 1:**
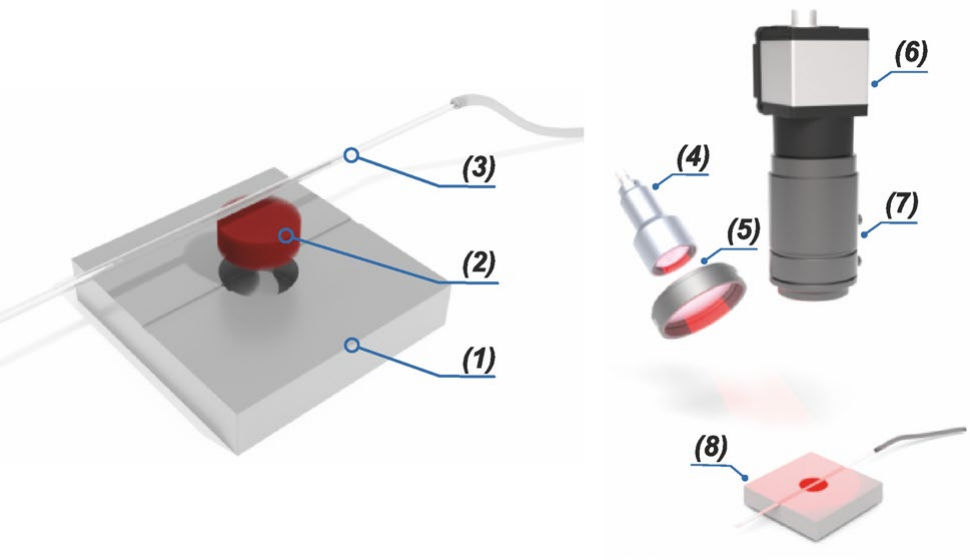
Schematics of the multi-component dynamic light scattering phantom of the mouse brain and skull (left): (1) – static base, (2) – porous media soaked in scattering solution simulating blood perfused brain tissues, (3) – glass capillary for simulation of the directed flow of large vessels; 3D layout of the LSCI setup used for the characterization of the phantom (right): (4) – coherent light source, (5) – optical diffusor, (6) – CMOS camera, (7) – objective lens (8) – phantom

The developed multi-component phantom of mouse skull and brain (see Fig. 1) consisted of three parts: a static base of the phantom imitating the static area with the optical properties of mouse skull bones (1), a porous medium filled with scattering solution designed to simulate blood-perfused brain tissues (2), and a glass capillary (3) simulating directed blood flow in a large vessel (e.g., sagittal sinus) of the brain.

The proposed 3-component structure of the phantom, combining a static scattering base, a diffusely perfused porous medium, and a directed flow channel, was designed to enable separate control and characterization of static regions, randomized, and unidirectional flow regimes. This approach is in line with recommendations for realistic flow phantoms in quantitative speckle-based imaging, as outlined by Qureshi et al. [36] who highlighted the need for independent simulation of different hemodynamic components to improve calibration and validation of optical flow measurement techniques.

The phantom base measures 40 × 40 × 8 mm and is made of a material with optical properties close to those of the mouse skull (see characterization of phantom properties below). At its center, the base features a cylindrical cavity with a diameter of 12 mm and a thickness of 5 mm. The base was fabricated using stereolithography (SLA) 3D printing with a UV-curing polymer resin (Formlabs Inc, USA). Transparent flexible UV resin (Formlabs Flexible 80A v.1) was used as the matrix for the base component. Scattering properties were achieved by incorporating zinc oxide nanoparticles (Sigma-Aldrich GmbH, Germany) with an average diameter of 0.34 µm at a concentration of 7.75 mg/ml. Absorption was controlled by adding black pigment (Formlabs Black (K)) at a concentration of 0.1% v/v.

**Fig. 2:**
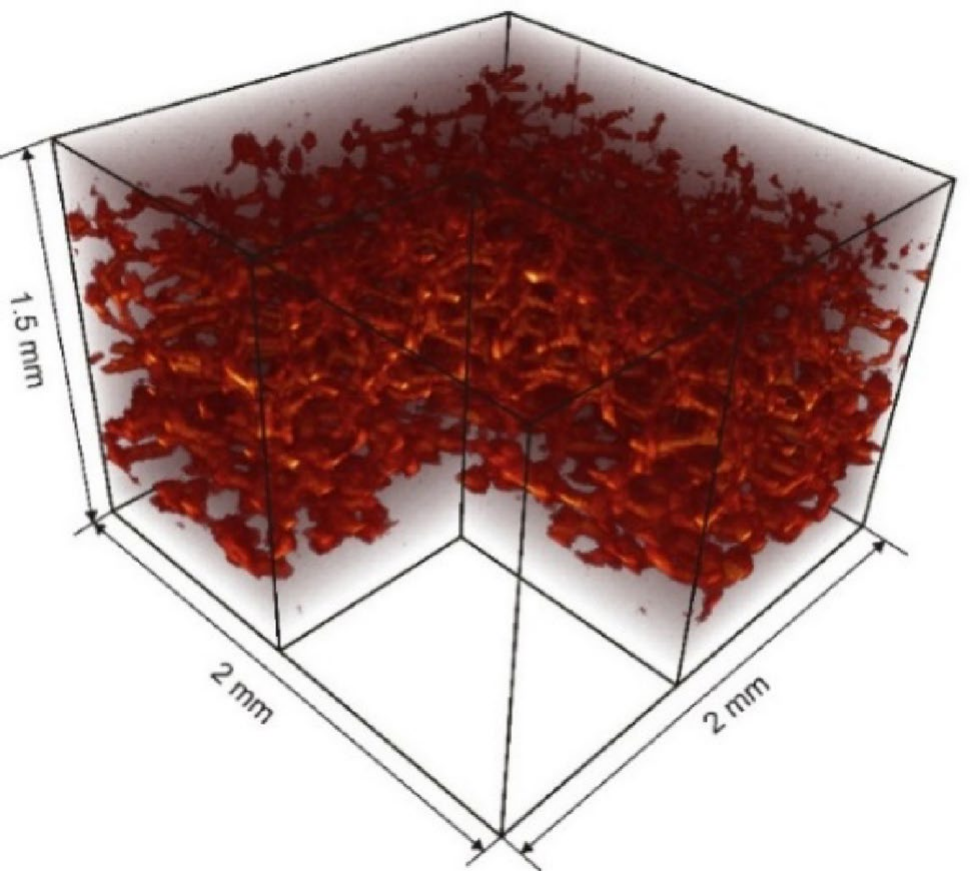
3D OCT image of melamine sponge demonstrates the open pore structure

The cavity is filled with a disc of fine porous material, precisely sized to fit, and infused with a blood-mimicking solution to replicate blood perfusion in brain tissues. The design focuses on the dynamic scattering properties and imitates the hemodynamics of small, randomly oriented vessels in the mouse brain. The porous material is capable of intake of a specific volume of scattering liquid, effectively simulating the desired conditions. Melamine foam with a sponge-like structure was chosen for this purpose. The sponge structure was characterized with an optical coherence tomography system (Thorlabs Ltd., Hyperion) at a central wavelength of 930 nm and an axial resolution of 5.8 µm (in air). Figure 2 displays the 3D OCT image of the material's structure, demonstrating its open pores architecture. The estimated average pore size of the sponge is about 100 µm. Due to its large pore size and open-pore architecture, the melamine sponge is assumed to possess superior liquid uptake capabilities. Additionally, the melamine sponge preserves its shape during the soaking process, preventing swelling or shrinking.

To induce dynamic light scattering, a 5% Intralipid solution was prepared by diluting Intralipid 20% (Sigma-Aldrich GmbH, Germany). Red dye (E120, Dr. Oetker AB, Sweden) was added to adjust the absorption spectrum of the melamine sponge and Intralipid solution, bringing it closer to that of blood. Two dye concentrations, 25% and 12.5% v/v, were evaluated. For both concentrations, the solutions were prepared to maintain a constant Intralipid concentration.

To prepare and install the sponge simulating blood-perfused brain tissue, a cylindrical disc of predetermined thickness was punched from a melamine sponge sheet using a sharp cylindrical punch and placed precisely into the well of the static base. The disc was then thoroughly saturated with a mixture of Intralipid and red dye to ensure complete impregnation. Excess liquid was carefully removed with a lint-free cloth. The well provided a tight fit without applying excessive pressure, ensuring the disc remained securely in place and maintained uniform contact with the surrounding phantom structure. Afterward, the soaked sponge was covered with a rectangular glass capillary with external/internal dimensions of 0.2 × 1.1/0.1 × 1.0 mm (see Fig. 1). The capillary was connected to a syringe of an infusion pump (Fusion 100, Chemix, USA) via flexible silicone tubes. The pump provided directed flow at a controllable rate, simulating blood flow in large vessels of the mouse brain [37]. The Intralipid–dye mixture at a concentration of 12.5% was continuously pumped through the capillary at flow rates ranging from 0 to 100 μl/min.

The directed flow channel in the phantom enables precise control of unidirectional flow rates, providing a platform to evaluate imaging sensitivity across a range of flow velocities. This capability is particularly relevant to speckle-based imaging methods, where the accurate detection of motion contrast depends on both flow magnitude and imaging parameter optimization, as demonstrated by Mariampillai et al. [38].

### Laser speckle contrast imaging setup

To estimate the dynamic scattering properties of the developed phantom, the LSCI technique was used in this study. Briefly, changes in the local dynamics of the observed medium (movement of scattering particles), cause fluctuations in the speckle pattern. As a result, the pattern becomes blurred in the captured frame due to the finite exposure time of the camera [39]. The faster the movement within the observed object, the more blurred the speckle pattern appears. This blurring leads to a decrease in the speckle contrast value, which can be used to quantify the flow rate. The speckle contrast parameter $K$ is defined as:

1 $$ K = \sigma /\left\langle I \right\rangle $$

where $\left\langle I \right\rangle$ is the mean intensity and $\sigma$ is the intensity standard deviation.

Fercher and Briers [40] further developed this equation and showed the relation between speckle contrast value, camera exposure time $T$, and speckle correlation time $\tau$ via the theory of correlation functions, which is widely used in DLS [41, 42].

2 $$ K\left( T \right) = \left[ {\frac{2\beta }{T}\mathop \smallint \limits_{0}^{T} \left| {\frac{{g_{1} \left( \tau \right)}}{{g_{1} \left( 0 \right)}}} \right|^{2} \left( {1 - \frac{\tau }{T}} \right)d\tau } \right]^{\frac{1}{2}} $$

where $K(T)$ is the speckle contrast depending on the camera exposure time. Fercher and Briers assumed that $\beta$ coefficient should be equal to 1. However, later it was shown that $\beta$ coefficient relates to the temporal field autocorrelation function ${g}_{1}(\tau )$ [43]. Also, it was further accepted that $1/{K}^{2}$ is an approximate estimation of $T/\tau$ which is related to real flow velocity [44–48]. The ratio $1/{K}^{2}$ is often called the blood flow index (BFI) [49] or speckle flow index (SFI) [50].

In the current study, the conventional LSCI setup has been used (see Fig. 1). Specifically, a 40-mW laser diode at 850 nm (RLDH850-40-3, Roithner Lasertechnik GmbH, Austria) was used as a light source. The light from the laser diode was further expanded using a diffuser (ED1-C20, Thorlabs, USA) to illuminate the investigated phantom. The speckle pattern reflected from the phantom was registered using CMOS camera (DCC3240M, 1280 × 1024, pixel size of 5.3 μm, Thorlabs, USA) in combination with a 12 mm F1.4 objective (Kenko Tokina Co., Ltd, Japan) at 7 ms exposure time. The obtained grayscale images were processed using a custom-developed algorithm in an offline regime within the MATLAB R2019a software environment. For the calculation of speckle contrast images, the spatial speckle contrast algorithm with the sliding window was used [21, 22]. For each measurement, 40 consecutive frames were captured. Speckle contrast images were calculated for each frame using a sliding window with a size of 7 pixels. Further, all these images were averaged to obtain the final speckle contrast image.

## Results and discussion

### LSCI characterization of the phantom

**Fig. 3:**
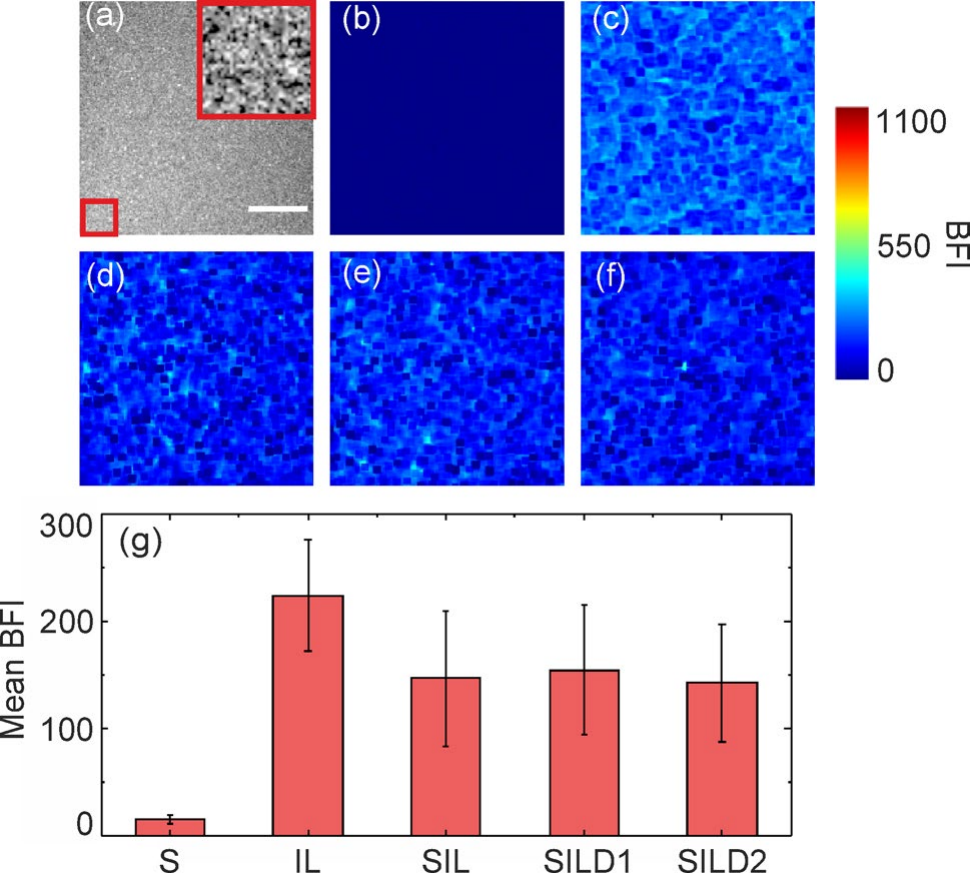
**a** Raw speckle image for dry sponge; **b** BFI images corresponding to dry sponge (S), **c** 5% Intralipid solution (IL), **d** sponge soaked in 5% Intralipid solution (SIL), **e** sponge soaked in a mixture of 5% Intralipid solution and 12.5% red dye (SILD1), **f** sponge soaked in a mixture of 5% Intralipid solution and 25% red dye (SILD2), **g** Corresponding bar diagram indicating the average BFI values. Error bars represent standard deviation. The scale bar is equal to 3 mm

The main components of the phantom were characterized through the LSCI measurements. Figure 3 shows examples of the obtained BFI images for a static dry melamine sponge (S), 5% Intralipid solution (IL), sponge soaked in 5% Intralipid (SIL), and sponge soaked in a mixture of Intralipid 5% with a red dye at different concentrations of 12.5% (SILD1) and 25% (SILD2).

**Fig. 4:**
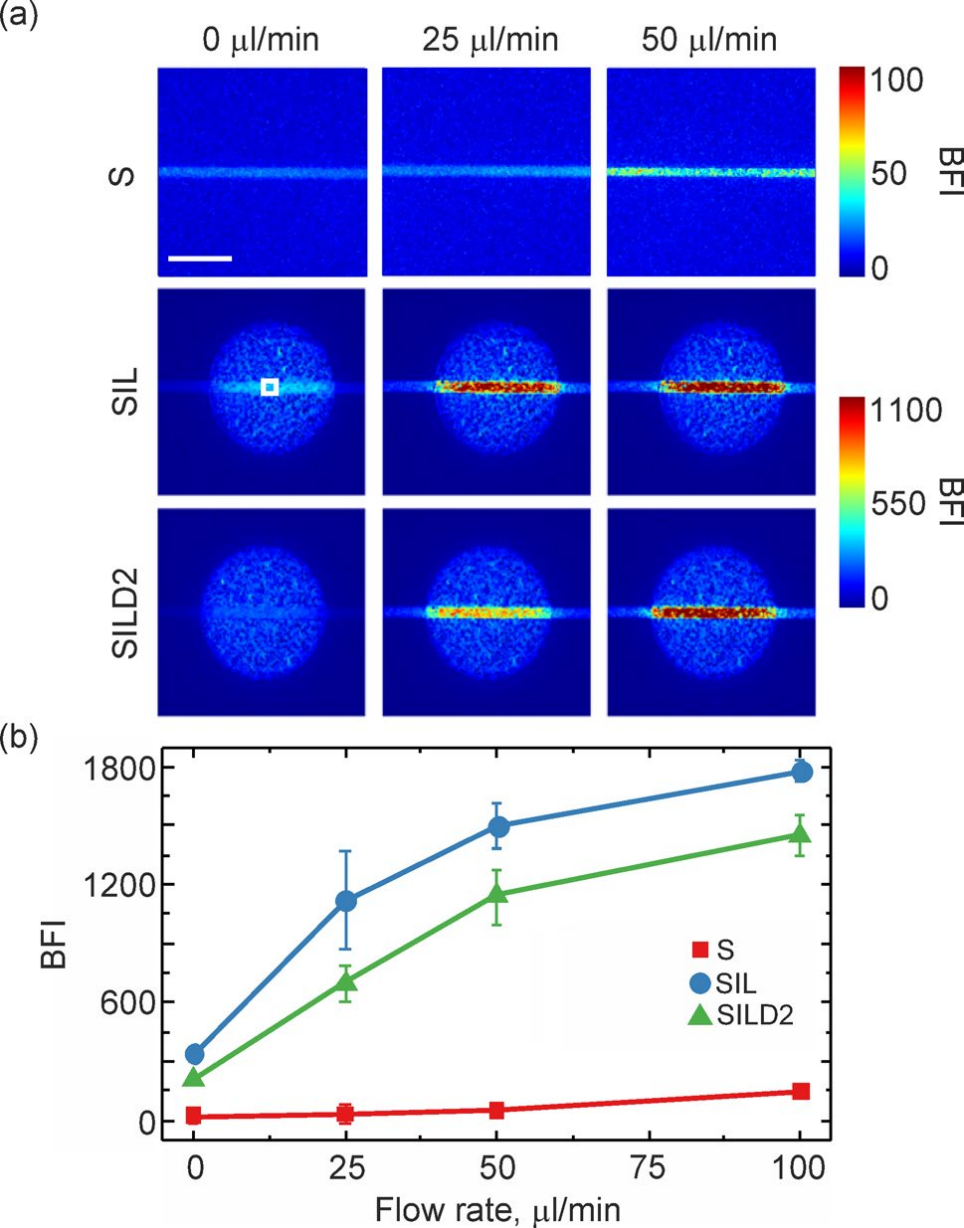
**a** BFI images for the assembled multi-component phantom (static base, sponge, and capillary) at different flow rates in the capillary. The measurements were performed for the sponge in S, SIL, and SILD2 states. **b** Averaged BFI values in the area corresponding to the vessel (white square in Fig. 4a) at different flow rates for the considered cases. The scale bar is equal to 3 mm. (Color figure online)

Figure 3g presents a bar diagram showing the average BFI values for the cases under consideration. In the images analyzed, lower BFI values are associated with static areas in the observed medium, such as the dry sponge. Conversely, the highest BFI values correspond to dynamic regions, exemplified by the Intralipid solution at Brownian motion. For the sponge phantom soaked in either the Intralipid solution (SIL) or the Intralipid–dye mixtures (SILD1–2), the BFI exhibits intermediate values, falling between the extremes of the completely static (S) and dynamic (IL) cases. These finding highlights that incorporating a melamine sponge effectively modulates the resulting BFI. Additionally, the sponge structure stabilizes the liquid component's behavior by mitigating meniscus-related issues and reducing the evaporation rate. Further, the assembled multi-component phantom, comprising a static base, a sponge (in S, SIL, and SILD2 states), and a capillary, was evaluated under flow conditions. The results of LSCI characterization are presented in Fig. 4a.

Figure 4b shows the mean BFI values calculated over the vessel area (see white square in Fig. 4a) for different flow rates and phantom states. Expectedly, it is seen that the estimated BFI value increases with the capillary flow rate increase. In addition, one can note that the SILD2 phantom has lower BFI values compared to the SIL phantom and is significantly higher compared to the dry sponge (S). Since the sampling volume for the used light source covers both the vessel and underlying layers of the phantom, the presence of a static component of the phantom under the vessel significantly affects/reduces the BFI [21]. In particular, the presence of the static base under the capillary reduces its BFI by several dozen times compared to IL cases (see Fig. 4b). In addition, for the flow range of 0 – 50 μl/min, the BFI values show a linear increase for the SILD2 case, while for the SIL case, the increase is essentially nonlinear.

### Comparison of the phantom and in vivo measurements

A comparative analysis was conducted between the BFI patterns obtained from the manufactured phantoms and those measured in vivo from the mouse brain. The raw speckle data for in vivo LSCI of mouse brains were sourced from our previous studies [27, 29]. Specifically, data for Mouse 1 (M1) were detailed in [27], while data for Mouse 2 (M2) and 3 (M3) were provided in [29]. For a comprehensive description of the animal preparation and experimental procedures, refer to [27, 29]. Shortly, the mice were anesthetized, the skin over the head was removed, and BFI measurements were performed transcranially at comparable experimental conditions using a laser source in NIR region (λ ~ 810 nm).

**Fig. 5:**
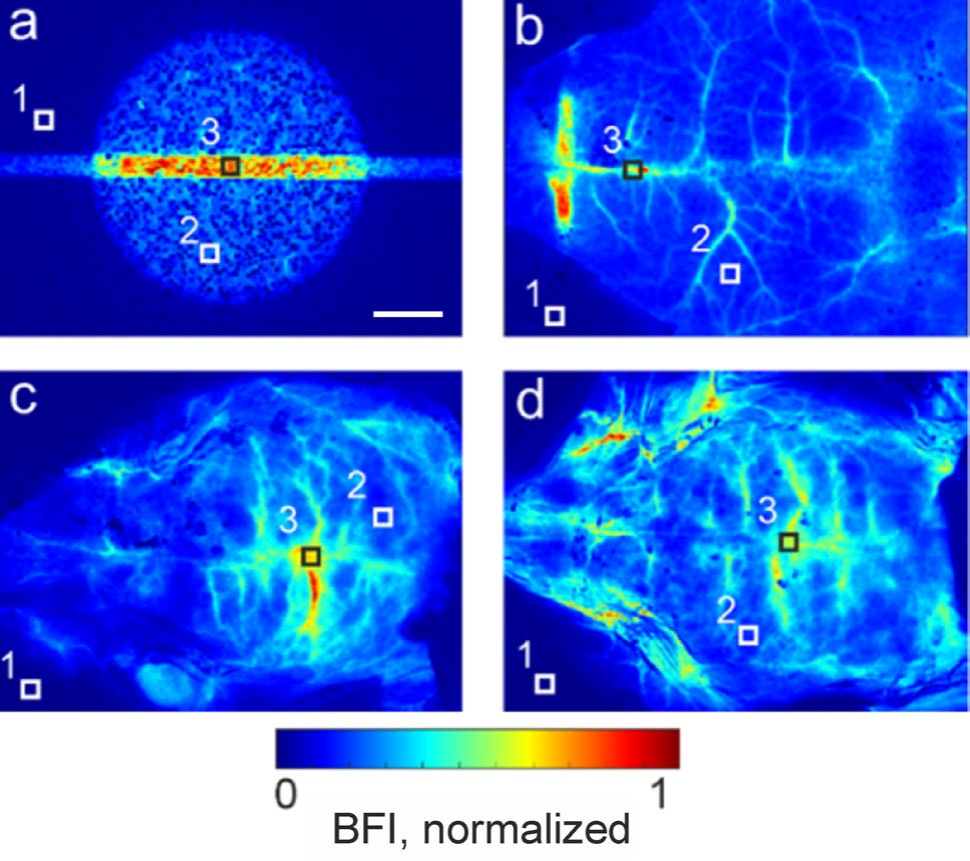
A comparison of normalized BFI images of the multi-component phantom of mouse brain (**a**) and in vivo mouse 1 (**b**), mouse 2 (**c**), and mouse 3 (**d**) brains imaged transcranially. 1 – static area, 2 – an area with blood perfusion, 3 – an area of the directed flow in a blood vessel. The scale bar is equal to 3 mm

Figure 5 presents typical BFI images, normalized to their maximum values, corresponding to both phantom and mice experiments. Each BFI image was analyzed by selecting three distinct types of regions: (1) a static area with no flow or perfusion, (2) a brain region perfused by blood in small, multidirectional vessels, and (3) a region containing a large vessel (sagittal sinus) characterized by directed blood flow in the mouse brain (the typical location of each region is shown by areas 1, 2, 3 respectively at Fig. 5 for each experiment).

For the comparison, the SILD2 phantom at the flow rate of 50 μl/min in the capillary has been selected due to the good correspondence between normalized BFI values of the phantom and in vivo measurements in all three selected areas.

**Fig. 6:**
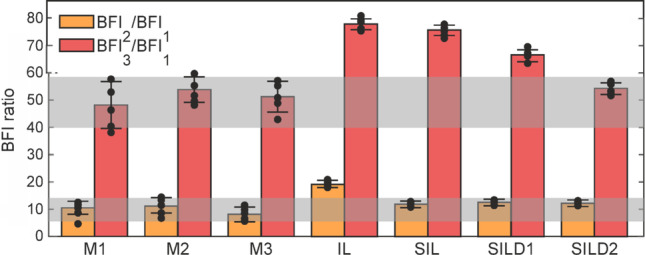
BFI values of dynamic areas (area with blood perfusion (2), area of the directed flow in a large vessel (3)) normalized to the BFI values of the corresponding static areas (1) for the in vivo measurements of mice 1–3 and considered phantoms

The quantitative comparison of mean BFI values for the selected types of dynamic in the in vivo mouse and proposed phantom are presented in Table 1 and Fig. 6. For a more robust statistical analysis, the mean ratios BFI_2_/BFI_1_ and BFI_3_/BFI_1_ were calculated across five distinct areas of the same type, each measuring 5 × 5 pixels. The resulting values are depicted as black dots in Fig. 6. Gray horizontal bars indicate the range of BFI ratio values that fall within the maximum and minimum standard deviations across all three mice.

**Table 1:** The average ratio between BFI values in dynamic areas (area with blood perfusion (2), area of the directed flow in a large vessel (3)) and in the static area (1) for Mice 1–3 and considered phantoms

Sample		BFI ratio of dynamic and static areas	
		BFI_2_/BFI_1_	BFI_3_/BFI_1_
In vivo	M1	7.1	45.5
	M2	9.5	43.4
	M3	9.9	47.6
Phantoms	IL	16	78.2
	SIL	10.9	76.9
	SILD1	10.8	58.2
	SILD2	10.6	48.4

Thus, as shown in Fig. 6 and Table 1, the good quantitative agreement between the developed phantom and hemodynamics of real mice measured transcranially by the LSCI method is demonstrated for the SILD2 configuration.

From Fig. 6, it is also seen that the increase in the attenuation coefficient (see e.g. SIL, SILD1-2 cases) lowers the measured BFI values corresponding to the same flow rates, which is in good agreement with research published earlier [51] before.

**Fig. 7:**
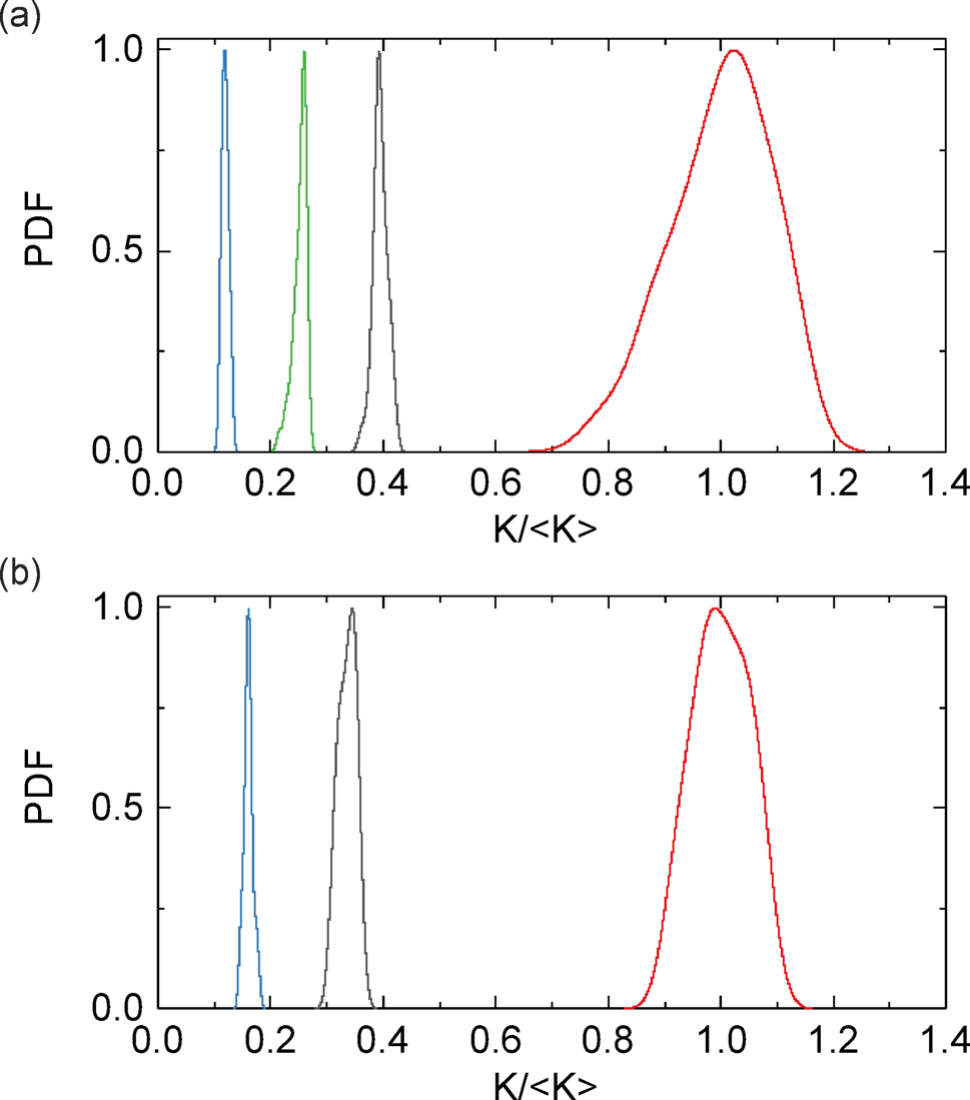
PDF corresponding to static area (red), area with blood perfusion (gray), area of the directed flow in a blood vessel (blue) – areas 1, 2, and 3 in Fig. 6, respectively. Measurements for SILD2 phantom (**a**) and M1 (**b**). Green curve shows PDF for pure Intralipid solution for comparison. (Color figure online)

Additionally, to characterize BFI and speckle contrast (*K*) values corresponding to different types of motion in animals and phantom, the probability density function (PDF) of the speckle contrast [52] was calculated for areas 1, 2, and 3 for the phantom and M1 experiment (see Fig. 7). Before PDF calculation, the measured *K* values for areas 2 and 3 were normalized to the *K* values in the static area 1. The PDFs for different areas (static, perfused, and directed flow regions) exhibit distinct distributions, that clearly indicate a statistical difference of speckle contrast in the considered cases. One can observe the effect of decrease in speckle contrast values jointly with the narrowing of their PDFs in both phantom and in vivo cases with increasing the movement velocity and directionality. In particular, the widest and highest distributions correspond to the static areas. The perfusion areas have narrower distributions and intermediate *K* values. The narrowest distribution and the lowest *K* values correspond to the directed flow area. The good agreement of normalized PDFs between phantom and in vivo data is demonstrated. This fact additionally supports the validity of the phantom for neurovascular studies. The obtained distributions clearly show the influence of both flow rate and optical attenuation on the speckle contrast values, providing a quantitative framework for optimizing phantom design.

### Characterization of the optical properties of the phantom

**Fig. 8:**
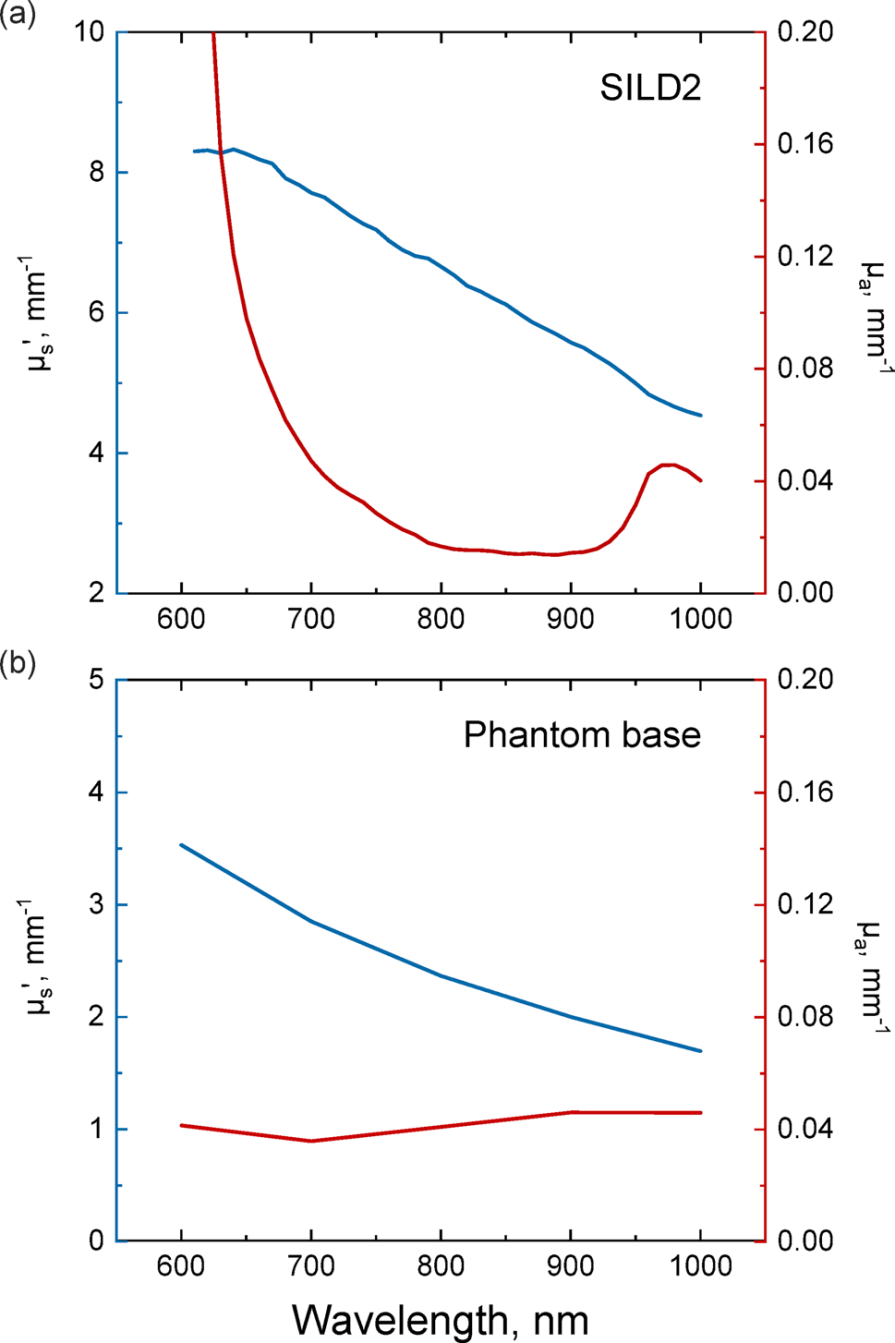
Reduced scattering and absorption coefficients of the SILD2 phantom component (sponge soaked in a mixture of Intralipid 5% and red dye 25%) (**a**); static base (**b**). (Color figure online)

Scattering and absorption properties of the SILD2 phantom were characterized with the integrating sphere spectrophotometer (OL-750, Optronic Laboratories, USA), in the range of 600–1000 nm. For this purpose, diffuse reflectance and transmittance were measured from the 1-mm-thick layers of the corresponding materials. The optical properties were retrieved using the inverse adding-doubling method. The details of the measurement approach have been previously described in [53]. The results of the measurements are presented in Fig. 8. The obtained optical properties are comparable with those of the mouse brain and skull [54, 55] at the measurement wavelength of 850 nm.

## Limitations

The observations during multiple imaging sessions indicated that the phantom maintained its structural integrity and consistent optical appearance over the day of use, provided that the melamine sponge remained hydrated and was not physically damaged. For long-term utilization of the phantom, it is important to note that the sponge component may degrade or lose uniformity if dried and rehydrated. Thus, for each new experimental session, it is recommended to use a freshly prepared melamine sponge cylinder to simulate blood-perfused brain tissue. The original melamine sponge material is highly uniform in structure, which allows for the consistent fabrication of cylindrical inserts with reproducible geometry and optical properties.

Our phantom is designed to model directed flow in a large cerebral vessel, such as the sagittal sinus, where physiological pulsations are minimal compared to arterial circulation. For this reason, we implemented a constant flow rate, which simplifies the setup, ensures precise control of experimental parameters, and remains representative of venous hemodynamics in such structures. We acknowledge that in other vascular compartments, particularly arteries, pulsatility from cardiac cycles can significantly influence hemodynamics and LSCI measurements. Incorporating pulsatile flow in future versions of the phantom, for example, by using a programmable pump to reproduce physiologically relevant waveforms, would extend the model’s applicability to arterial conditions and broaden the scope of in vivo validation.

Although the melamine sponge provides a convenient porous structure for simulating microvascular perfusion. Its pore geometry does not directly replicate the architecture of cerebral capillaries. The distribution and orientation of the pores are random and do not follow the organized vascular patterns of brain tissue. However, a full replication of small capillary geometries was not the goal of this study. Instead, the aim was to find a stable and reproducible medium that could generate diffuse, randomized flow and optical scattering similar to that observed in perfused tissue. A more detailed comparison with in vivo microvascular parameters could still be useful in future work to further assess anatomical relevance and guide potential material modifications for applications requiring higher physiological precision.

The proposed phantom can also be suitable for a range of optical imaging techniques that rely on similar to LSCI contrast mechanisms, e.g., Diffusion Correlation Spectroscopy (DCS), Laser Doppler Flowmetry (LDF), and speckle variance OCT (svOCT).

## Conclusion

This work presents a multi-component dynamic light scattering phantom designed to replicate the complex hemodynamics of the mouse brain by incorporating both static and dynamic components. A melamine sponge soaked in a 5% Intralipid solution combined with a 25% concentration of red dye was found to be an adequate model of the blood-perfused brain tissue, providing a more physiologically relevant representation of cerebral microcirculation. The experimental results demonstrate a strong quantitative agreement in terms of BFI values between the developed phantom and in vivo transcranial measurements of the mouse brain, validating its effectiveness as a model for optical imaging studies.

The BFI index of the perfusion area was shown to be highly dependent on both the structural inclusion of the sponge, which mimics the microvascular network of the brain and the optical attenuation properties of the blood-mimicking liquid. The porous structure of the sponge effectively stabilizes the dynamic scattering properties, allowing for better control of the perfusion dynamics compared to Intralipid solution alone. The inclusion of red dye enables fine-tuning of attenuation characteristics, bringing the BFI values closer to those observed in biological tissue.

Similarly, the BFI index of the directed flow region was found to be controllable through both the flow rate and the optical attenuation of the medium. The study confirmed a linear increase in BFI with increasing flow rates up to 50 µl/min for the SILD2 phantom. Furthermore, the presence of an absorbing medium reduced BFI values in the large vessel, emphasizing the role of optical attenuation in controlling the flow index. Our findings show the importance of combining static and dynamic components as well as the need for careful selection of scattering and absorbing agents to achieve realistic optical modeling of cerebral hemodynamics. The proposed phantom provides a tool for the calibration and validation of DLS-based neuroimaging techniques and offers an accurate and reproducible platform for investigating cerebral blood flow dynamics in preclinical research.
